# Supplementary material for: Dual-responsive Gemini Micelles for Efficient Delivery of Anticancer Therapeutics
Source: Polymers (Basel). 2019 Apr 2;11(4):604. doi: 10.3390/polym11040604 (PMC6523983; doi:10.3390/polym11040604)

Supporting information

Dual-responsive gemini micelles for efficient delivery of anti-cancer therapeutics

Young In Choi,[a,†] Eun-sook Choi,[a,†] Kwan Ho Mun,[b] Se Guen Lee,[a] Sung Jun Lee,[a] Sang Won Jeong,[a] Eunjoo Kim,[a] Seung Woo Lee,[b,*] and Hyun-Chul Kim [a,*]

[a] *Convergence Research Institute, Daegu Gyeongbuk Institute of Science and Technology (DGIST), Daegu 42988, Republic of Korea.*

[b] *School of Chemical Engineering, Yeungnam University, Gyeongsan 38541, Republic of Korea*

Figure S1. Fluorescence spectra with DOX concentrations in DMF (A) and calibration curve obtained from standard DOX solutions (B).

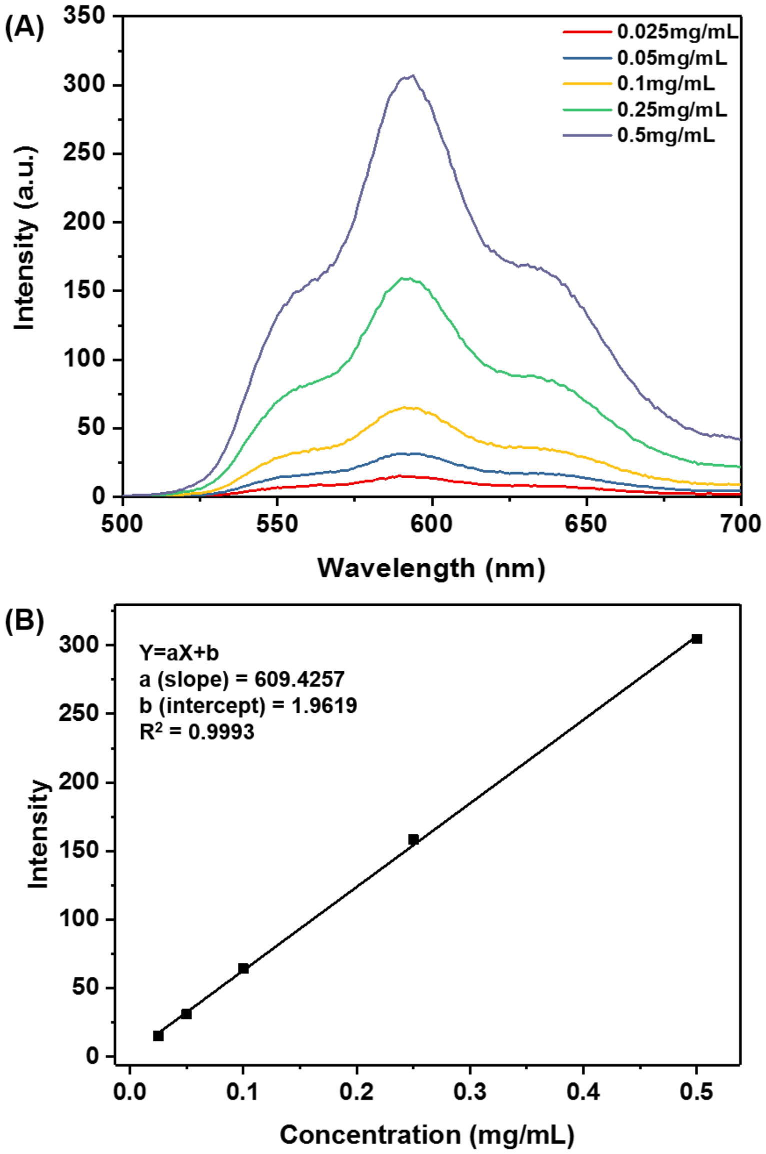

Figure S2. GPC traces of monomeric mPEG-Cys-PMT and gemini mPEG-Cys-PMT measured by using THF as a mobile phase.

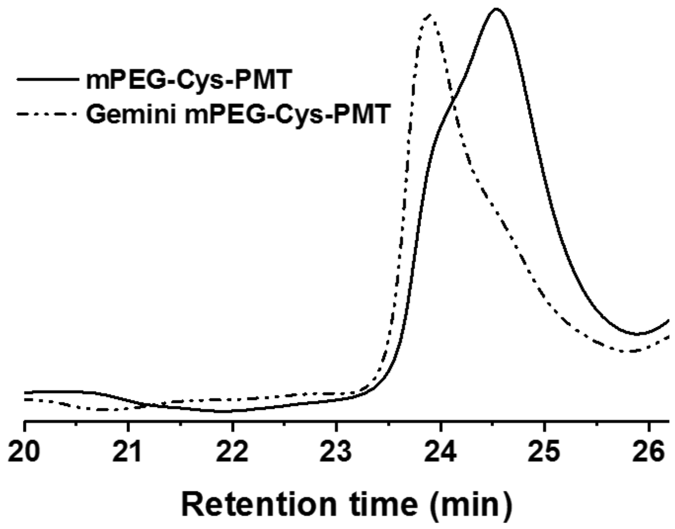

Figure S3. Plot of the intensity ratio I_{337}/I_{333} from the pyrene excitation spectra versus the logarithm of (A) monomeric mPEG-Cys-PMT and (B) gemini mPEG-Cys-PMT treated with 10 mM DTT.

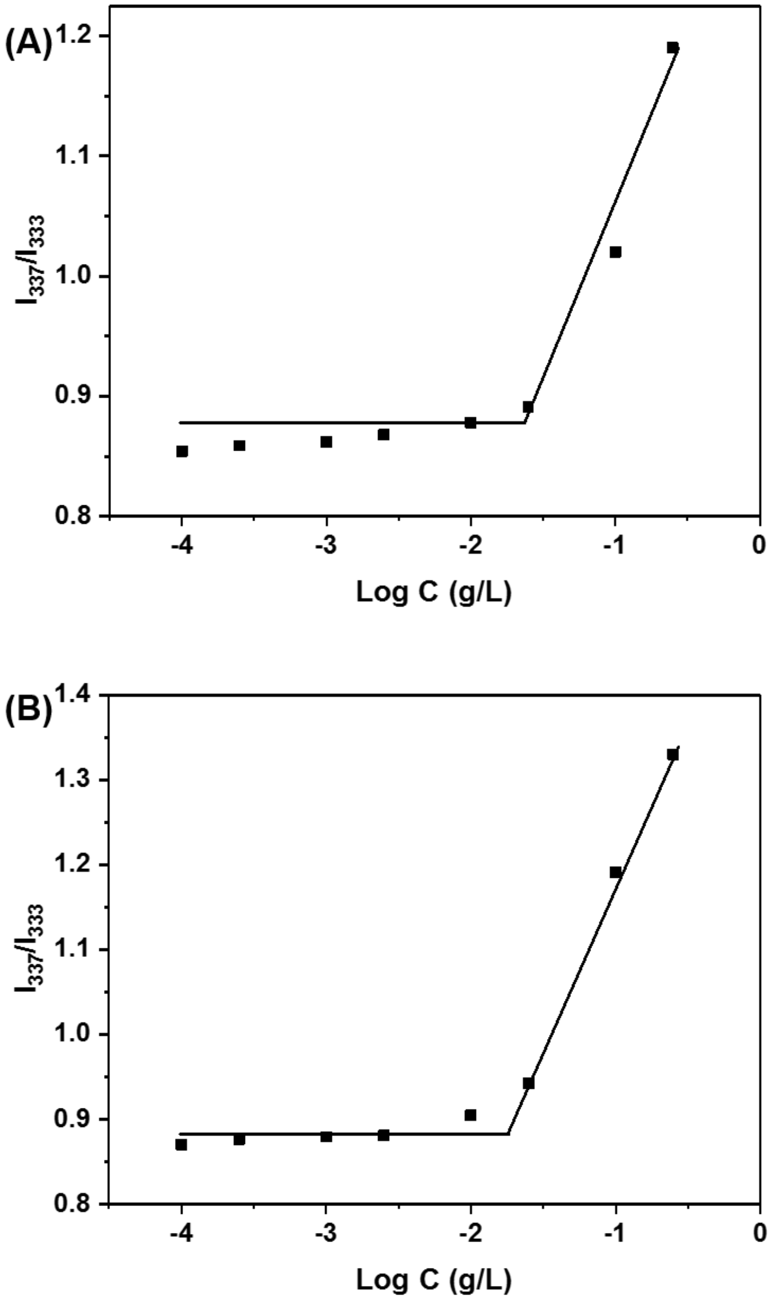

Figure S4. Drug loading contents and efficiency of gemini mPEG-Cys-PMT micelles with feed weight ratios of polymer and DOX.

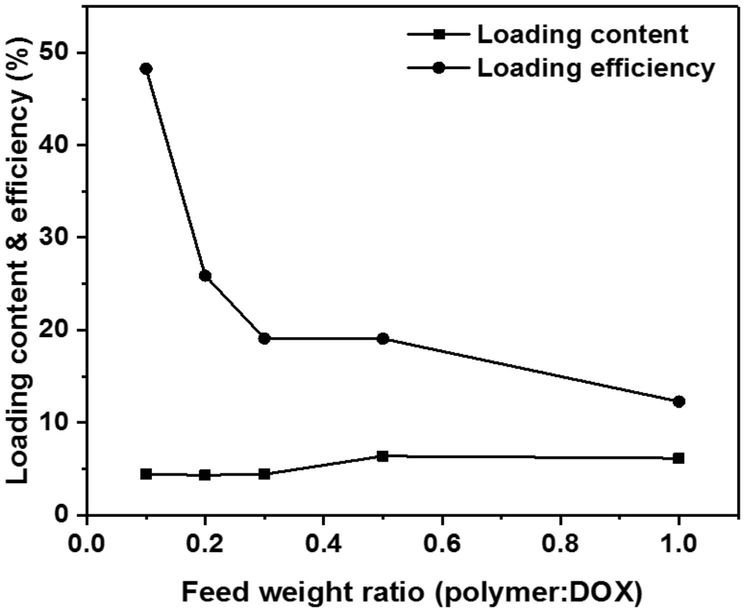

Supplement: Supplementary file 1 [file polymers-11-00604-s001.pdf]
